# Establishing trust in emergency telehealth consultations

**DOI:** 10.1111/1742-6723.14543

**Published:** 2024-12-03

**Authors:** Jennie Hutton, Veal Michael, Suzanne M Miller, Belinda Baines, Marija Kirjanenko, Loren Sher, Joanna Lawrence, James Boyd, Adam Semciw, Rebecca Jessup, Jason Talevski

**Affiliations:** ^1^ Victorian Virtual Emergency Department The Northern Hospital, Northern Health Melbourne Victoria Australia; ^2^ School of Psychology and Public Health La Trobe University Melbourne Victoria Australia; ^3^ Department of Critical Care, Melbourne Medical School The University of Melbourne Melbourne Victoria Australia; ^4^ Allied Health, The Northern Hospital, Northern Health Melbourne Victoria Australia; ^5^ Plunkett Centre for Ethics Australian Catholic University Sydney New South Wales Australia; ^6^ School of Exercise and Nutrition Sciences Institute for Physical Activity and Nutrition Research (IPAN), Deakin University Geelong Victoria Australia; ^7^ Victorian Virtual Specialist Consult Service The Northern Hospital, Northern Health Melbourne Victoria Australia; ^8^ Victorian Centre for Virtual Health Research The Northern Hospital, Northern Health Melbourne Victoria Australia

**Keywords:** doctor–patient relationship, emergency, telemedicine, trust

## Abstract

Over recent years, emergency telehealth has developed rapidly in Australasia. From the patient's perspective, establishing trust with a healthcare provider is uniquely challenging when using the audio and video modalities commonly used in telehealth. It is crucial to consider how we may improve the delivery of care through this emerging pathway if high‐quality care is to be delivered. Several simple techniques have been identified in the literature and can be employed to create trust and improve the patient–provider relationship. These include ensuring privacy and an appropriate setting for the consultation; considering how eye contact and expressions are best used; providing alternative options to telehealth; and clearly identifying names, roles and qualifications. We describe how these methods can best be employed in the virtual emergency care setting.

Emergency telehealth has developed as a means to effectively and safely divert patients requiring urgent healthcare away from EDs, while offering patients care at a time and place of their convenience.^1^, ^2^ The success of this model of care relies on both patients and healthcare providers having a clear and mutual understanding of the consultation process, including how to relay, interpret and action recommendations made during the consultation. Emergency telehealth consultations may be vastly different to the patient's previous experiences with traditional ‘in‐person’ healthcare. However, unlike many telehealth consultations, emergency telehealth is provided by a clinician who is has most likely not met the patient previously. In Australia, if patients are enrolled in Medicare, some or all the cost of seeing a general practitioner is covered by Medicare. However, to receive telehealth payment benefits, the patients must have an existing relationship with the doctor and have had a previous in‐person consultation within 12 months.^3^ For virtual emergency care, a single unplanned consultation is undertaken, often in the stressful context of an acute illness or injury. This form of consultation presents a new challenge for emergency clinicians who seek to ensure patients feel they can trust the provider and are satisfied with their care.

Trust can be defined as ‘the expectation that healthcare providers will do good for their patients, recognising the vulnerability of patients in the unequal nature of the patient–provider relationship’.^4^ Patients who have higher levels of trust in their healthcare provider report greater satisfaction with treatment; exhibit more positive health behaviours; experience fewer adverse symptoms; and show improvements in quality of life.^5^ Trust fosters open communication, ensures patients feel heard and valued and enhances confidence in the care received.^6^ In addition, understanding how trust develops in telehealth has been recognised as a patient‐centred research priority for clinicians and academics.^7^

There has been a large body of literature exploring the establishment of trust in healthcare interactions for face‐to‐face consultations. Studies have identified that physical touch plays a key role in fostering a trusting patient–provider relationship.^6^, ^8^ During a virtual emergency consultation, alternative communication strategies need to be identified and utilised to address the limitations of the absence of a physical presence. With this in mind, this paper aims to report on factors and strategies that may improve trust in a virtual emergency model of care.

## Presenting alternative care options

Presenting telehealth as an option rather than the sole method of accessing healthcare can empower patients, and act as an enabler to shared decision‐making, potentially enhancing the trust between patient and clinician.^9^ A study by Graham *et al*. on paediatric rehabilitation delivered via telehealth showed families were more likely to trust in the technology when healthcare providers presented it as one option among others, rather than the only available mode of care.^9^ When telehealth was framed as the sole option, patients felt undervalued by clinicians and perceived the virtual interaction as less valuable than face‐to‐face care. Training clinicians to discuss all available treatment and consultation options using clear communication and consent is encouraged, emphasising patient choice. This will enable shared decision‐making between patients and healthcare providers.

## Consult setting

Emergency telehealth changes the setting of a consultation away from a hospital or clinic to the location of the patient's choice. For many patients, this results in greater comfort, as they are seen in a more casual and familiar environment, which can improve their trust in telehealth.^10^ For these patients, meeting their provider in a comfortable environment also increases their sense of autonomy. This is particularly important for groups that have experienced previous trauma in face‐to‐face emergency care settings, such as First Nations peoples, people with disabilities, and those with mental health disorders.^11^ However, finding a private and comfortable space for a telehealth consultation may be challenging for individuals living in shared accommodation or unstable housing. A systematic review of the telehealth literature highlighted the importance of an appropriate setting for developing a strong patient–provider relationship.^12^ An initial assessment by the clinician of the patient's privacy should be undertaken, asking if the patient is in a private and comfortable space. This will ensure confidentiality, which is critical to establishing trust in a telehealth consultation.^13^ For individuals without stable housing, telehealth service providers could partner with community centres or public libraries to offer private rooms equipped for virtual consults.

## Qualifications

Patients have expressed a preference for viewing the qualifications of their healthcare providers before starting a consultation.^14^ This transparency assures patients that they are consulting with an experienced and qualified provider, thereby increasing their trust in the remote care being offered. In the absence of traditional physical cues indicating a clinician's role, the practitioner should clearly self‐identify their role through both written (e.g. displayed on screen) and verbal communication, if possible, at the beginning of the consult. This approach is comparable to hospital settings, where clear identification of clinicians through name badges can improve the quality of care and patient satisfaction.^15^ Trust is considered by some as a more relevant indicator of quality care than patient satisfaction alone.^16^

## Eye contact

Eye contact plays a pivotal role in helping patients feel a genuine connection with their healthcare provider, despite the physical distance.^6^, ^17^ Helou *et al*.'s study focused specifically on eye contact in telemedicine, using pre‐recorded videos of clinicians to study patient perceptions. The study highlighted that in videoconferencing, mutual gaze is an illusion achieved by looking into the camera rather than the eyes of the other participant onscreen. Patients perceived physicians as making more eye contact when they looked directly into their cameras, rather than at the patient's image on the screen.^18^ Conversely, when physicians did not make eye contact, patients perceived other aspects of care quality to decrease, including empathy to show understanding of the patient's feelings, expressing concern, and willingness to help.^19^ However, this result was only obtained when physicians had been instructed to come across as tired and distractable; when physicians were trying to be more caring and attentive, eye contact did not impact these perceptions. It is also important to consider each care episode individually, as the appropriateness of eye contact may vary based on cultural background, emotional state, personality traits, and gender of both the patient and clinician. Eye contact may be amplified by a close‐up screen in virtual healthcare, which can sometimes make eye contact seem intrusive or aggressive, so it must be tailored to the individual.^20^ Providing clinicians with practical tips, such as adjusting their screen setup or employing adjunct devices that enable the impression of eye contact could improve the connection between the patient and provider.

## Establishing a video presence

Establishing a strong video presence is pivotal to keeping patients engaged and ensuring they feel they are receiving high‐quality telehealth care.^12^ In the telehealth context, exaggerating non‐verbal communication can be useful and beneficial. An exaggerated effect can ensure non‐verbal cues such as facial expressions and gesticulations are clearly understood over video (e.g. a gentle nod can indicate understanding, agreement or encouragement).^12^, ^21^ Similarly, longer pauses can be necessary because of potential delays in the audio feed.^22^ When patients feel they are adequately heard, they are likely to communicate any concerns; ask clarifying questions; and provide additional details if asked.^6^ Fluid responses without any unnecessary breaks or pauses can also demonstrate empathy. Furthermore, maintaining sustained engagement from a child during paediatric consultations is challenging. Finding unique ways to engage children of variable ages is imperative to maintaining interest, with puppets, magic tricks and toys being a useful tool for this.^23^ At the same time, for patients with dementia, reducing background noises and allowing for longer consults might be helpful strategies to use.

## Conclusion

Trust is a cornerstone of effective telehealth care because it directly influences patient engagement, satisfaction and outcomes. In the absence of a physical presence, establishing trust between the patient and healthcare provider becomes even more crucial to providing high‐quality emergency telehealth care. In Australia's multicultural society, further investigation is required in how to best cultivate trust for different communities using telehealth. This is essential in delivering culturally acceptable, safe and accessible care. In particular, focusing on building trust with First Nations communities has the potential to strengthen relationships with emergency healthcare services and improve healthcare access. Establishing a patient feedback system to continuously evaluate and enhance telehealth experiences is crucial for optimising service efficacy, acceptability and the overall development of this emerging healthcare delivery model. It is essential that both individual clinicians and services actively seek feedback from patients to facilitate ongoing improvements and add to this limited body of knowledge. Several strategies to improve the telehealth process have been identified in the literature and can easily be employed by clinicians working in the emerging field of emergency telehealth.

### Competing interests

None declared.
